# Experimental Study to Evaluate the Wear Performance of UHMWPE and XLPE Material for Orthopedics Application

**DOI:** 10.3390/bioengineering9110676

**Published:** 2022-11-10

**Authors:** Sandeep Bhoi, Arbind Prasad, Ashwani Kumar, Rudra Bubai Sarkar, Bidyanand Mahto, Chandan Swaroop Meena, Chandan Pandey

**Affiliations:** 1 Department of Mechanical Engineering, Parala Maharaja Engineering College, Berhampur 761003, Odisha, India; 2 Department of Mechanical Engineering, Katihar Engineering College (Under Department of Science & Technology, Government of Bihar), Katihar 854109, Bihar, India; 3 Technical Education Department Uttar Pradesh, Kanpur 208024, Uttar Pradesh, India; 4 Research and Development Department, Tata Steel Ltd., Burma Mines, Jamshedpur 831007, Jharkhand, India; 5 Government Engineering College Vaishali (Under Department of Science & Technology, Government of Bihar), Vailshali 844115, Bihar, India; 6 CSIR-Central Building Research Institute, Roorkee 247667, Uttarakhand, India; 7 Academy of Scientific and Innovative Research (AcSIR), Ghaziabad 201002, Uttar Pradesh, India; 8 Mechanical Department, Indian Institute of Technology, Jodhpur 342030, Rajasthan, India

**Keywords:** UHMWPE, Cross Link PE, tribological, abrasive wear, biomedical, orthopedics

## Abstract

The main objective of this study is to perform an abrasive wear resistance study of UMHWPE and XLPE by using different grades of abrasive paper (grade 100 (190 µm), grade 220 (50 µm), and grade 400 (40 µm)) with minor (10 N) and major (15 N) loading conditions. In this article, wear performance of the UMHWPE and XLPE materials compared to the bio-tribological data as reported earlier in the clinical studies has been investigated. The experimental result shows that the loss of materials for the XLPE was much higher than the UHMWPE under similar loading conditions. UHMWPE shows a 34% reduction in wear at minor loading conditions and a 53% reduction in wear at major loading conditions. From experimental results it was concluded that Cross-link PE has better wear resistance than UHMWPE in minor wear conditions, whereas UHMWPE shows better wear resistance under major loading conditions. Based upon these results, UHMWPE and XLPE have been recommended for use as bearing materials in orthopedics. The experimental results of this study were validated using results from the available literature.

## 1. Introduction

In the human body, different joints play many important roles, and they provide rotation, twist and turn motions to the joining body parts. Due to unavoidable circumstances, i.e., ageing, accidents or sudden loading from a person being overweight causes damage to the body joints, which requires advance biomaterials for joint healing or replacement. Biomaterials are used for many biomedical applications such as total hip replacement (THR) and total knee replacement (TKR). Wear investigations of these joining surfaces for artificial hip joints and knee joints play a significant role in reducing wear particle formation, extending the implant’s lifetime and avoiding revision surgeries. Tribological mechanism studies in artificial joints, integrated studies of friction, wear rate, and lubrication. Biomaterials such as ultra-high-molecular-weight polyethylenes (UHMWPE) are mostly used in orthopedics for artificial joints as a bearing material. Increasing the cross-links in polyethylene (XLPE) material leads to improvement in wear performances. These characteristics of UHMWPE and XLPE materials have been examined in this research article using a wear study. 

Biomaterials aid in extending human longevity and quality of life, and the field of biomaterials has seen significant expansion to meet the needs of an ageing population. Composite biomaterials are used in several parts of the human body, including prosthetic heart valves, blood vessel stents, and replacement implants in the shoulder, knee, hips, elbows, and ears, as well as for several other orthopedic applications [1,2,3,4]. Bone is considered as a natural biocomposite material that consists of hydroxyapatite and collagens. Biomaterials can be used for different orthopedic applications such as scaffold fabrication, bone fixation devices, and orthopedic implants [5,6,7,8]. In joint replacement, total hip arthroplasty (THA) and total knee arthroplasty (TKA), is commonly performed to improve the functionality of load-bearing joints and increase patient mobility. Improved patient outcomes have prompted the expansion of total hip arthroplasty and total knee arthroplasty, with much of this success owed to the development of innovative biomaterials that are more durable, withstand greater force, and are more cost-effective. Total hip and knee replacements have seen a particularly large rise in occurrence. More importantly, prosthetists are now able to customize implants to meet the specific needs of each patient. 

Resorbable polymers have many advantages over metallic implants, but when there is a case of load bearing application then conventional biocompatible polymers play a vital role [9,10,11]. However, resorbable polymeric composites lack the mechanical strength and are widely used for internal fixation devices such as screws, plates, pins etc. to support bone fractures. In the case of knee joints and hip joints, wear is most seen near the joints and leads to problems in the cartilage, ligaments and tendons. If wear is high then the patient may feel extreme pain, which subsequently leads to bone fracture or related infections [12]. Bone-related diseases including osteoporosis (bone diminishing), osteoarthritis (irritation of the bone joints), and general injury are on the whole reasons for joint replacements. Not just have the replacement but the case of total hip and knee replacement also increases. Revision surgery is extremely painful and expensive as well. It was observed that the revision surgery of hip and knee were enhanced very much in past one decade. It is believed that 90% of the population beyond the age of 40 years experiences these kinds of degenerative infections, and the older population has continued to increase, with projections of a seven-fold growth within next one decade. Musculoskeletal issues are the most well-known human wellbeing concern, costing society more than 254 billion dollars around the globe [13,14,15]. 

Thus, in the coming days, the requirement of the implants is huge and therefore so is the demand for the biomaterials. To prevent the loosening of the implant, the properties of the biomaterials must lie in the range of bone properties so that stress shielding does not take place and thus the biomaterial closer to the bone must be applicable for bone implants [16]. 

## 2. Recent Developments in Biomechanical Tribology

In orthopedics, UHMWPE is frequently utilized as a load bearing material in a variety of artificial joints. Both THA and TKA [17,18] have been proven to cause osteolysis when worn in vivo, resulting in aseptic loosening. XLPE has been developed as an alternative to traditional UHMWPE, particularly in THR. The physical and mechanical properties of polyethylene, along with its wear resistance, have been considerably altered as a result of such cross-linking [19,20,21]. Cross-linking refers to the method involved with expanding the quantity of cross-links between the polymer chains of UHMWPE through repeated gamma sterilization cycles in nitrogen. Repeated nitrogen sterilization doses increase the gel content of UHMWPE, implying enhanced cross-linking. Strain hardening is observed in the irradiation-cross-linked UHMWPE [22]. The massive deformation mechanical properties at the articulating surface are closely related to the wear behaviour of irradiation-cross-linked UHMWPE. Since cross-connecting caused a huge reduction in flexibility and durability, the improvement in wear conduct ought to be considered with regard to other required mechanical characteristics. 

The huge deformity of mechanical responses of UHMPE under multi-axial loading circumstances has been linked to wear at the articulating surfaces of complete hip replacements, according to researchers. Cross-linking reduces the material’s hardness, toughness, and elastic modulus, prompting some authors to express worries about its long-term durability [23]. Loss of material (typically owing to contact), abrasion, adhesion, fretting, delamination, pitting (fatigue), and strains are the main causes of wear in total joint replacement. These factors affect friction, lubrication, contact area, surface polish, and load levels. When the atomic forces between two materials in contact are greater than the inherent property of each material bonding or fracture of asperities; wear debris is created and adhesive wear will be formed. Surface roughness is crucial because UHMWPE adheres to the counter bearing surface. In UHMWPE, it is linked to plastic flow [24]. Due to multi-axial stress conditions in the hip, a “plasticity induced damage layer” associated with plastic strain generates submicron debris. Abrasive wear occurs when surfaces of varying hardness come into contact with each other. The softer material is ploughed through by asperities on the tougher surface.

The wear rates of UHMWPE cups were generally higher according to retrieval tests. The wear rate of UHMWPE cups is affected by the concentration of PMMA particles in the lubricant as well as the femoral head material [25,26,27]. Polyethylene wear resistance has been improved through cross-linking. When compared to normal UHMWPE, it has a reduced penetration rate. Cross-linking has been shown in wear simulator experiments to lower the type of wear that occurs in acetabular components by more than 95%. Laboratory wear simulations, on the other hand, may not be indicative of in vivo performance. Clinical trials using XLPE are thus necessary to confirm the findings of in vitro studies. The clinical volumetric wear rate for XLPE was 81 % lower than that of conventional polyethylene, with a 72 % reduction in wear per million cycles, according to the results of an in vivo investigation. Creep and wear are responsible for the femoral head’s entire penetration into the acetabular polyethylene. The creep characteristics of conventional and cross-linked polyethylene have been found to be equivalent in recent laboratory experiments. However, despite the lower wear rate, concerns have been raised that the lower average size of the XLPE wear particles might lead to osteolysis [28,29]. 

**Figure 1:**
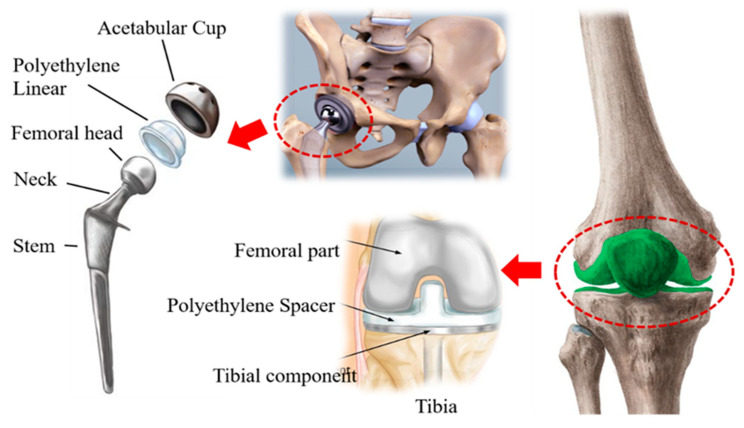
Schematic diagram showing Total Hip Replacement (THR) and Total Knee Replacement (TKR) [29].

The biomaterials associated with natural resources may be useful in restoring and regenerating the functions of the undeveloped structures. Figure 1 shows the location of biomaterials to be used in total hip replacement and total knee replacement.

In recent years tremendous work has been done in biomaterials and researchers have used experimental, numerical, FEA simulation and RSM optimization tools for different mechanical and biomedical analysis. In continuation Wang et al. [30] have studied the UHMWPE wear properties using PMMA particles for hip joint heads in simulator. Bhoi et al. [31] have studied the tribological properties of low carbon steel materials and Patil et.al. [32] have studied the tribological study of PIB and PMA. Kumar et al. [33] have performed the thermal contact conductance using an FEA simulation tool. Gangwar & Kumar et.al [34,35,36,37,38] studied the femur and humerus bone using various boundary conditions. They have studied fracture failure and natural frequency variations for the femur and humerus bones. They have also used advanced materials for fracture healing and orthopedics applications. For biomedical analysis and optimization these researchers have used advanced tools like FEA & RSM. These tools have been used widely in mechanical and materials engineering for fracture to vibration analysis [39]. 

In present study authors have used experimental method for the abrasive wear resistance of UMHWPE against XLPE was investigated experimentally in minor (10 N) and major (15 N) loading conditions. During experiments, different grades of abrasive paper and with minor and major loads has been applied and a comparison of the wear performance of the above two materials with clinical studies has been investigated here. UHMWPE is a favorable material for orthopedic and spine implants due to load and impact bearing capacity. The clinical results of XLPE are outstanding, but problems related to strength as well as crosslink stability persist [40,41]. Thus, wear characteristic assessments are necessary, and these materials can be suggested for clinical applications under different conditions. 

## 3. Experimental Procedure and Problem Definition

**Figure 2:**
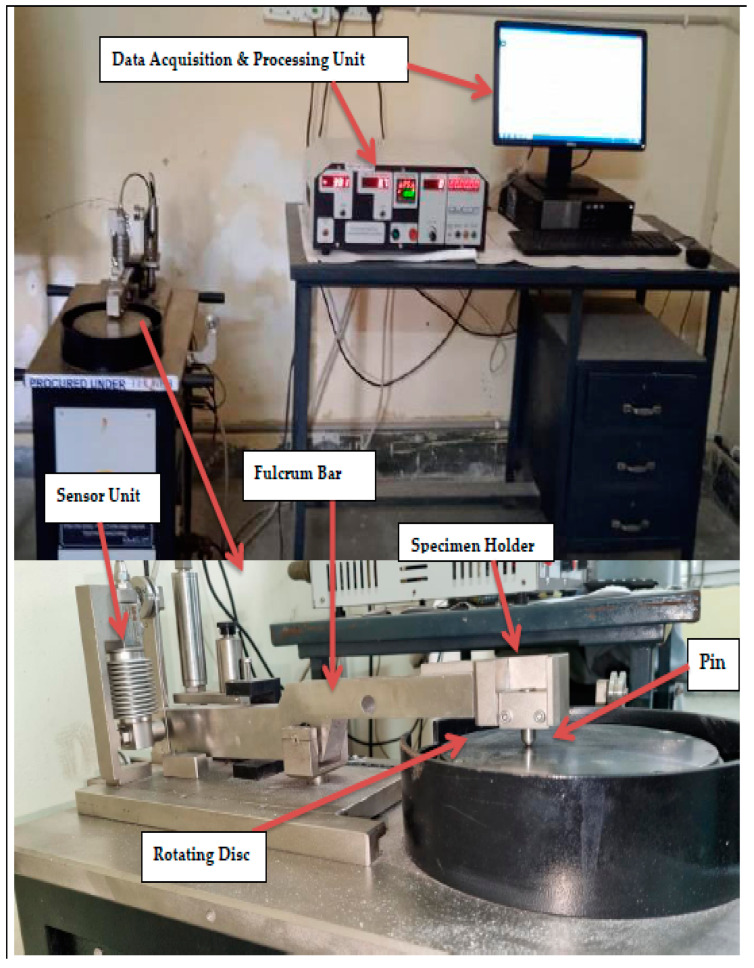
Schematic diagrams of experimental setup and enlarged view of pin-on-disc contact for wear analysis.

Tribological research on load-carrying interfaces for artificial hip joints is essential for decreasing wear particle formation and extending the life of the implant. To acquire a better knowledge of the complicated tribological mechanism in artificial joints, integrated studies of friction, wear, and lubrication are required. The creation of prosthetic hip joints has been aided by the application of tribological principles. The experiment was conducted in a pin-on-disc machine whose diagram is shown in Figure 2. The specimen holder has three degrees of freedom. It can translate in the horizontal plane along x and y axes and can rotate as shown in Figure 2. The position of the specimen holder is so adjusted that the maximum sliding distance is achieved for each rotation of the disc. The abrasive testing machine is set up for experimenting. It is cleaned thoroughly to remove dust, dirt, oil, or grease. The abrasive paper of the desired grade number is cut in the size of the rotating disc and is firmly secured to the disc, and minor (10 N) and major (15 N) loading conditions were applied experimentally. The specimen is cleaned with acetone, dried, its weight is noted with the help of a weighing machine [31], and its properties are listed in Table 1.

The specimen was fixed in the specimen holder, placed over the disc made up of reference material with abrasive paper. A load of 10 N is added to the machine. The machine is switched on and after 10 rotations of a disc, and the machine is turned off. The specimen is taken out and the loss in weight was noted. For the second experiment, a fresh abrasive paper of required grade was placed over the disc for testing. The cycle was repeated until we achieved the desired amount of sliding distance of the specimen over a given grade of abrasive paper. The specimens used for conducting abrasive wear tests are ultra-high molecular weight polyethylene and cross-link polyethylene. Three different grades of paper were used; they are of grade 100 (190 µm), grade 220 (50 µm), and grade 400 (40 µm). The load considered for this experiment was minor (10 N) and major (15 N). The specimens were made in the cylindrical shape of 35 mm in length and 10 mm in diameter. The abrasive papers were available in a standard size of the rectangular shape of a dimension of 280 × 230 mm. The abrasive papers were cut into a diameter of 178 mm to place over the rotating disc, as the diameter of the disc is 178 mm. The results obtained for both the specimens with three different grades of paper under load 10 N are presented in the form of a graph.

## 4. Results and Discussions

**Figure 3:**
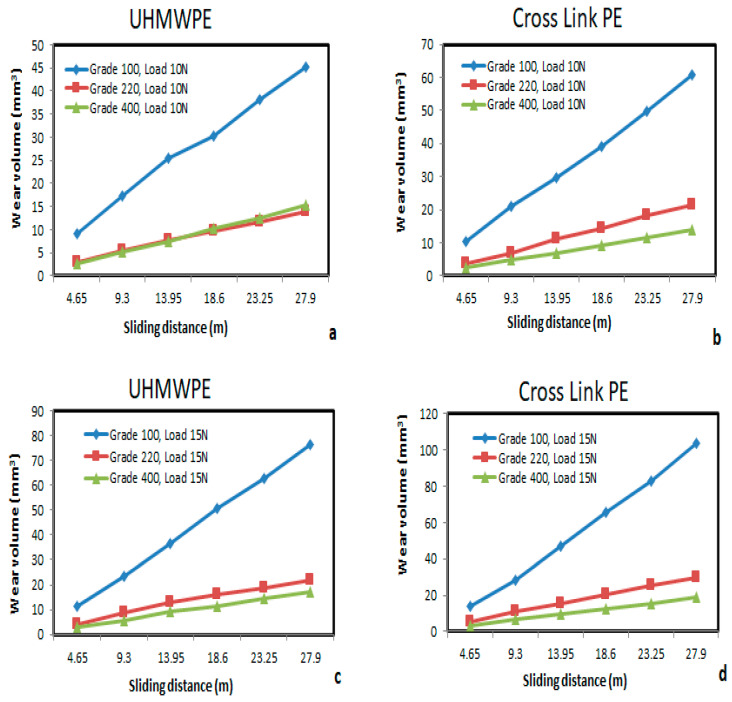
(**a**–**d**) Wear volume v/s sliding distance for UHMWPE and Cross Link PE at 10 N and 15 N loads with different grades of abrasive for wear test.

The wear behaviors of UHMWPE and Cross Link PE under different grades of abrasive papers with 10 N and 15 N (minor and major) loads were studied extensively. The wear volumes of both these biomaterials were plotted in the Figure 3a–d. To study wear characteristics, 100, 220 and 400 grades of abrasive papers with varying sliding distances were applied. From the Figure 3a,c (for UHMWPE), it can be observed that there is no significant changes while using 220 and 400 grades processed abrasive paper during test. The possible reason is that the interaction of biomaterial with higher grade abrasive paper will be smoother, as the grain size of 220 and 400 grade paper is less compared to 100 grade abrasive paper. Due to this, the wear volume is increasing with the increase in sliding distances under lower grade abrasive paper. It was observed that as the sliding distance increases from 4.65–27.9 m, the wear volume increases from 8–45 mm^3^. In this condition, specimen was wear against 100 grade abrasive paper having coarse structure. 

Similar wear behavior can also be observed in the case of Cross Link PE, as shown in Figure 3b,d. However, the interaction of this biomaterial with higher 220 and 400 grade abrasive paper exhibits certain differences which were not observed in the UHMWPE test. Under lower grade 100 abrasive paper, both of these biomaterials almost showed similar increasing trends (Figure 3a–d). 

Loss in material due to the wear test shows that material loss is more in XLPE under 10 N loads and the sliding distance variation is 4.65–27.9 m. There is a difference of 34% wear volume rate between these biomaterials when considering the 100 grades and 10 N load conditions. This difference increases to 53% under 220 grade abrasive, but it is reduced to 9% with 400 grade abrasive paper under the same load. 

**Figure 4:**
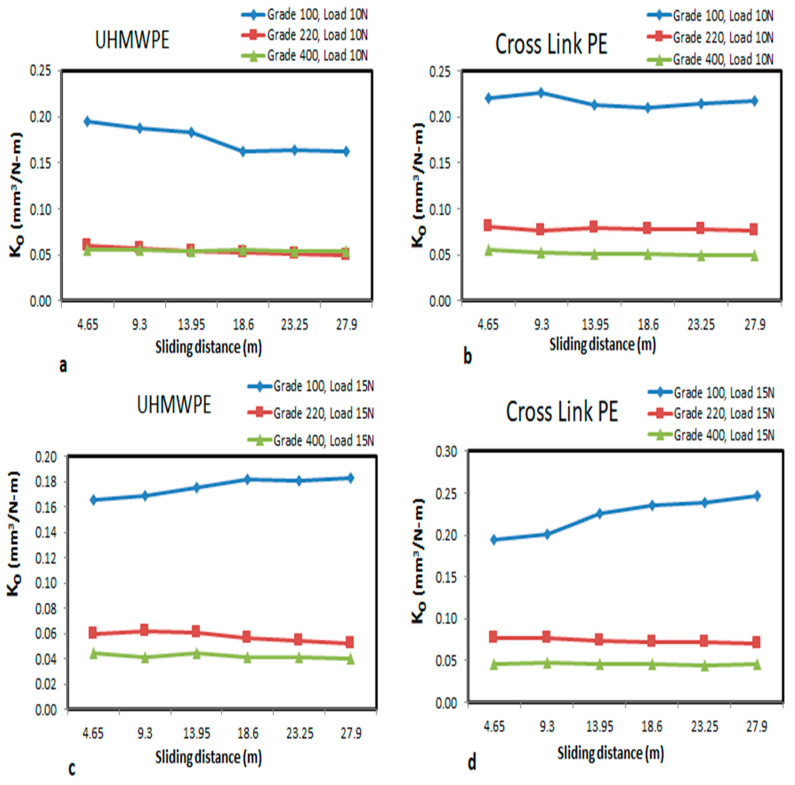
(**a**–**d**). Specific wear rate v/s sliding distance for UHMWPE and Cross Link for 10 N and 15 N loads with different grades of abrasive for wear test.

The specific wear rate can be calculated for both UHMWPE and Cross Link PE materials. The same is plotted against sliding distance with different grades of abrasive papers under the 10 N and 15 N load conditions, as shown in Figure 4a–d.

The specific wear rate in Figure 4a,c shows that almost negligible differences were observed with increasing sliding distance 4.65–27.9 m under 220 and 400 grades of abrasive. However, with 100 grade and a 10 N load condition, the specific wear rate of UHMWPE decreases with increasing sliding distances. This means that the material is getting stiffer as the rubbing distances increases. 

The specific wear rate of Cross Link PE shown in Figure 4b,d also exhibits similar behavior as that of the UHMWPE. However, in case of Cross Link PE, the specific wear rate is constant for different grades of abrasive papers with the change of sliding distances. This indicates that an increase in sliding distances will have less impact on the specific wear rate of Cross Link PE. Whereas on comparison with UHMWPE, the specific wear rate of Cross Link PE will have higher value for both 100 and 220 grade abrasive papers at a 10 N load. Due to the refined grain structure of 400 grade abrasive paper and the high stiffness of UHMWPE, Cross Link PE shows a less specific wear rate at the same 10 N load condition.

The co-efficient of wear ‘K’ is calculated by using the formula

V = K × (W/H)(1)

In Equation (1), the symbol V is the wear rate in volumetric wear/ sliding distance (mm^3^/mm). W is load in N and H is Vickers hardness number in MPa or N/mm^2^. 

The wear rate and wear coefficient calculated values are tabulated in Table 2. By varying the abrasive grade papers and the wear coefficient value range from 1.280 to 4.157 (at 10 N minor load) and 1.030 to 4.682 (at 15 N major load), for UHMWPE materials. However, in case of Cross Link PE, it ranges from 1.370 to 6.030 (at 10 N loads) and 1.270 to 6.870 (at 15 N loads). 

**Figure 5:**
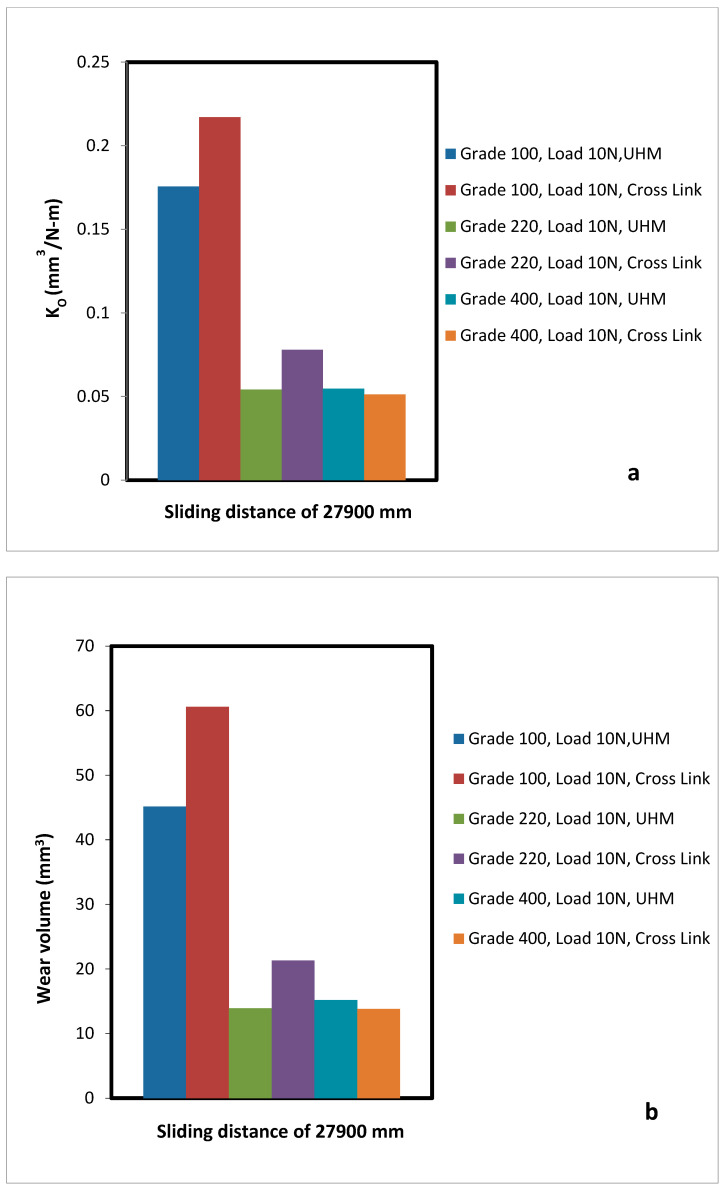
Full Comparison of UHMWPE and Cross-link PE (**a**) Specific wear rate at all wear conditions with load 10 N. (**b**) Wear volume at all wear condition with load 10 N.

**Figure 6:**
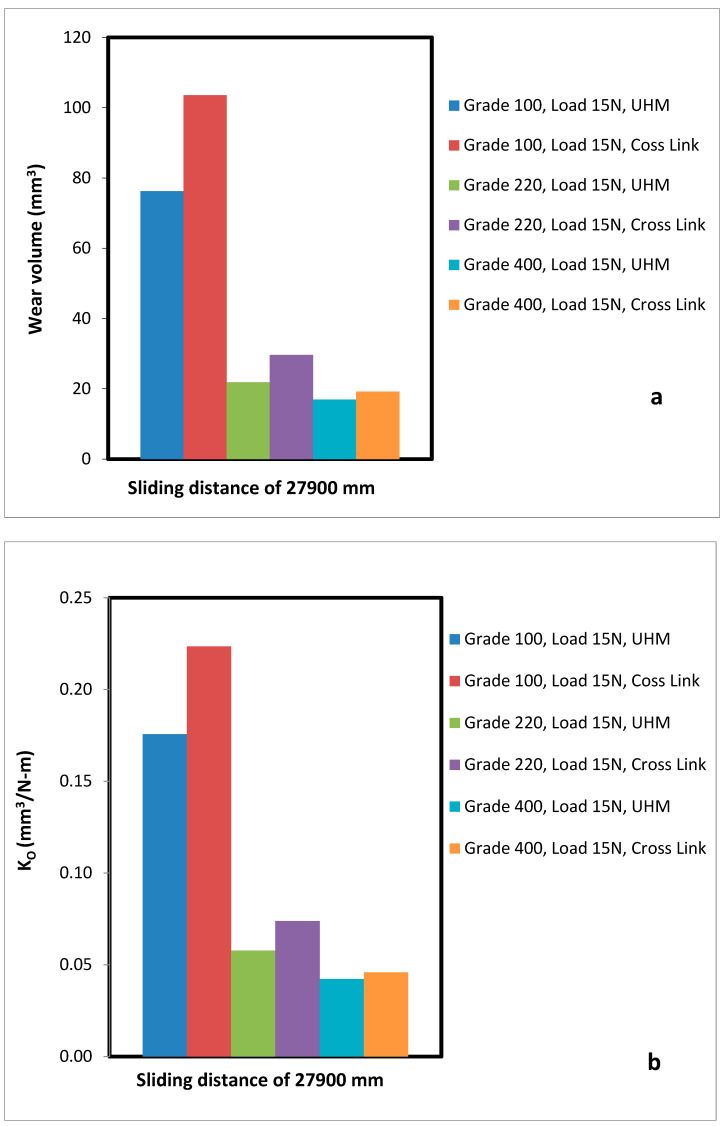
Full Comparison of UHMWPE and Cross-link PE (**a**) wear volume at all wear condition with load 10 N (**b**) Specific wear rate at all wear conditions with load 15 N.

A comparative analysis of wear volume and specific wear rate with a sliding distance of 27,900 mm was analysed between the UHMWPE and XLPE as shown in Figure 5a,b and Figure 6a,b. The Cross-Link PE material has a higher amount of material loss compared to UHMWPE. This demonstrates that Cross-Link PE is softer than the UHMWPE in terms of two body abrasion wear.

The above results of Figure 5a,b and Figure 6a,b can be validated using the available literature experimental results of John et al. [40]. They have performed a similar study to evaluate the wear characteristics of UHMWPE for hip joint applications. They have found the wear rate of UHMWPE to be 12.5 mg per million cycles, which can be converted to 19.1 mm^3^ per million cycles. In this experimental study the wear rate of UHMWPE was observed as (15.2–19.85) mm^3^ per million cycles when a wear test was conducted corresponding to the 220 and 400 grade abrasives. The maximum wear rate has a difference of 1 mm^3^ per million cycles. The deviation of results is less than 4%, which is an acceptable range. This is due to loading parameters and other configurations which have an effect on wear rates. 

### Micrographic Examination (SEM Test)

**Figure 7:**
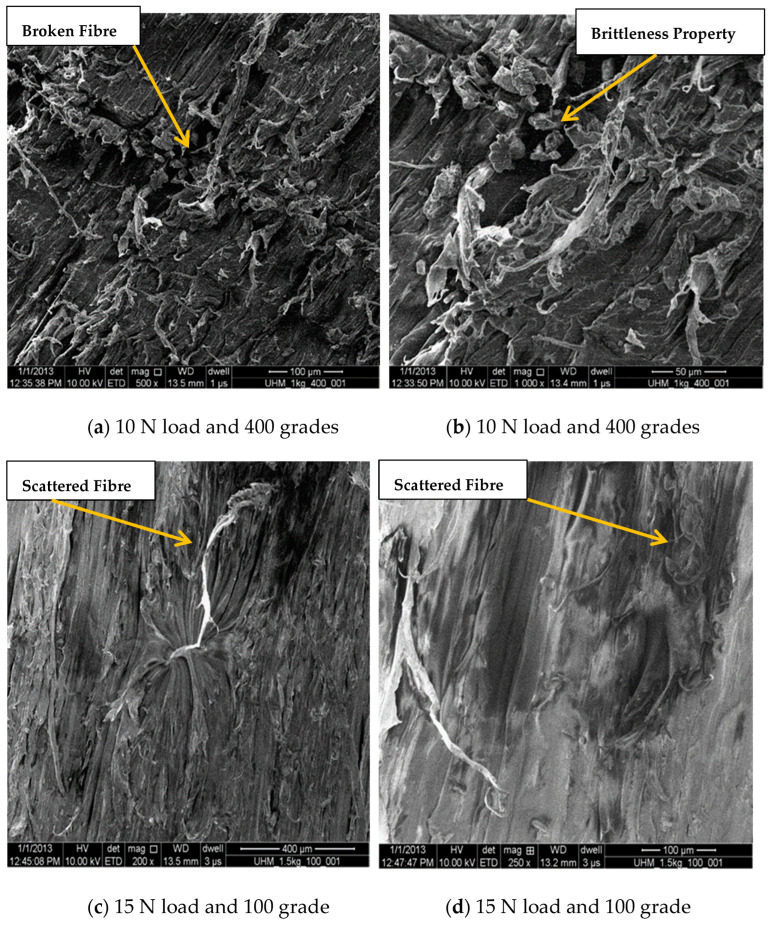
SEM analysis of UHMWPE at different load and grade conditions.

The micrographic examination was obtained through scanning electron microscope (SEM) images of UHMWPE after abrasion with 100 and 400 grade abrasive paper at 10 N and 15 N loads, as shown in Figure 7a–d. The brittle nature of UHMWPE can be clearly seen in Figure 7b, as scattered or broken fibers can be found in both the 100 µm and 50 µm magnification images. 

**Figure 8:**
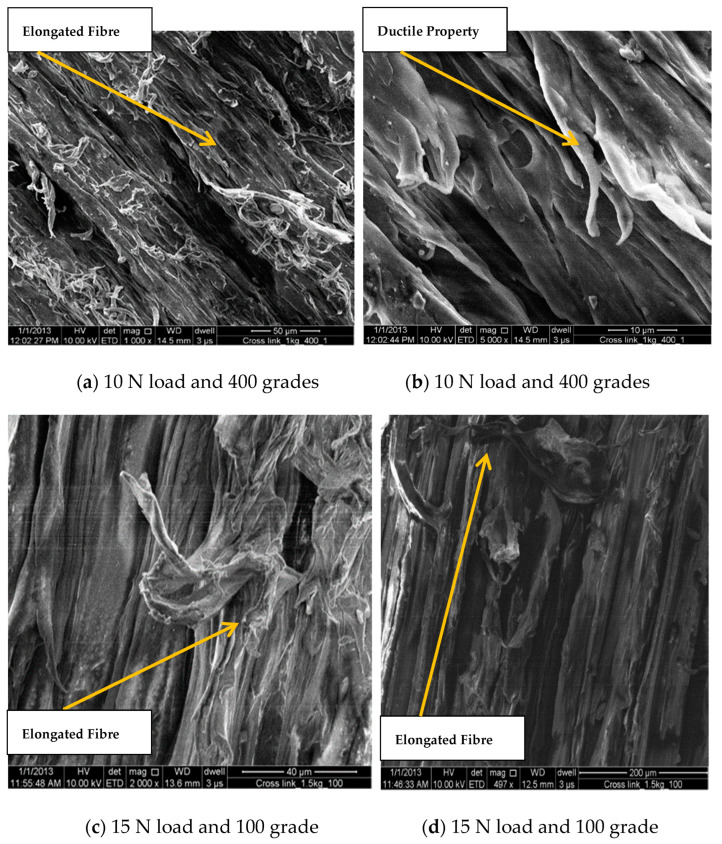
SEM analysis of XLPE at different load and grade conditions.

On the contrary, the ductile nature of Cross Link PE is being observed on the same load and with the same grade abrasive paper as the fibers are in elongated form (as shown in Figure 8a–d. Ploughing marks can be clearly visible on both the micrographs. This indicates the same ductile behavior of UHMWPE at 100 grade and 15 N major load condition. Similar scanning electron micrographs have taken for Cross Link PE as shown Figure 8c,d. In both figures ploughing marks can be seen, and a few micro cracks can be observed. The biomaterial is highly deformable at 100 grade and 15 N load conditions.

## 5. Conclusions

Earlier results of in vivo studies demonstrated that the clinical volumetric wear rate for cross-linked polyethylene was 81% lower than that for conventional polyethylene. However, in the present experimental study, it is found that Cross-link polyethylene has better wear resistance than UHMWPE, only with 10 N minor loads and fine grain size abrasive paper of grade 400. At major load conditions (15 N), i.e., a coarse grain size of abrasive paper i.e., 220 or 100 grade with UHMWPE shows better wear resistance than Cross-link PE. Thus, it was concluded that Cross-link PE shows better wear resistance properties at lower load conditions. However, in major load conditions, UHMWPE has greater wear resistance than Cross-link PE. One possible reason for this could be a reduction in toughness and an increase in brittleness of XL polyethylene material due to an increase in cross-linking. Due to this instability, cross-linked polyethylene shows poor wear resistance as compared to UHMWPE under major loading. 

It is recommended to use UHMWPE for orthopaedic applications such as with the femur, humerus bone fractures, and total hip and knee replacements due to its high wear resistance and high load carrying capacity. XLPE can be used for supporting internal fixation devices such as screws, plates and pins due to its wear resistance properties at minor loading. In future this research work can be extended to study the cross linked UHMWPE properties for higher load spine applications. 

## Figures and Tables

**Table 1:** Specimen Material Properties.

Material	Density (kg/m^3^)	Vickers Hardness Number (Hv)
UHMWPE	935	25.7
Cross-link PE	934	27.8

**Table 2:** The value of K for both materials under various wear conditions.

Grade	Material	Load (N)	Wear Rate (mm^3^/mm)	Wear Co-Efficient ‘K’ ×10^−3^
100	UHMWPE	10	1.617 × 10^−3^	4.157
100	UHMWPE	15	2.733 × 10^−3^	4.682
220	UHMWPE	10	4.98 × 10^−4^	1.280
220	UHMWPE	15	7.82 × 10^−4^	1.330
400	UHMWPE	10	5.44 × 10^−4^	1.390
400	UHMWPE	15	6.05 × 10^−4^	1.030
				
100	Cross L PE	10	2.17 × 10^−3^	6.030
100	Cross L PE	15	3.71 × 10^−3^	6.870
220	Cross L PE	10	7.63 × 10^−4^	2.120
220	Cross L PE	15	1.06 × 10^−3^	1.970
400	Cross L PE	10	4.95 × 10^−4^	1.370
400	Cross L PE	15	6.86 × 10^−4^	1.270

## Data Availability

Not applicable.
